# Characterization of the binding pattern of human aquaporin-4 autoantibodies in patients with neuromyelitis optica spectrum disorders

**DOI:** 10.1186/s12974-016-0642-3

**Published:** 2016-07-01

**Authors:** Friederike Tuller, Hannah Holzer, Kathrin Schanda, Fahmy Aboulenein-Djamshidian, Romana Höftberger, Michael Khalil, Thomas Seifert-Held, Fritz Leutmezer, Thomas Berger, Markus Reindl

**Affiliations:** Clinical Department of Neurology, Medical University of Innsbruck, Innsbruck, Austria; Department of Neurology, Karl Landsteiner Institute for Neuroimmunological and Neurodegenerative Disorders, Sozialmedizinisches Zentrum Ost Donauspital, Vienna, Austria; Institute of Neurology, Medical University of Vienna, Vienna, Austria; Department of Neurology, Medical University of Graz, Graz, Austria; Department of Neurology, Medical University of Vienna, Vienna, Austria

**Keywords:** Neuromyelitis optica spectrum disorders, Aquaporin-4, Autoantibodies, Epitope specificity, Flow cytometry

## Abstract

**Background:**

The discovery of a highly specific antibody against the aquaporin-4 (AQP4) water channel (AQP4-IgG) unified the spectrum of neuromyelitis optica spectrum disorders (NMOSD), which are considered to be antibody-mediated autoimmune diseases. The AQP4 water channel is located on astrocytic end-feet processes and consists of six transmembrane helical domains forming three extracellular loops A, C, and E in which defined amino acids were already proven to be critical for AQP4-IgG binding. However, the clinical relevance of these findings is unclear. Therefore, we have characterized the epitope specificity of AQP4-IgG-positive NMOSD patients.

**Methods:**

We established a cell-based flow cytometry assay for the quantitative detection of AQP4-IgG-positive serum samples. Human embryonic kidney (HEK) cells were transiently transfected with an EmGFP-tagged AQP4-M23, AQP4-M1, or six AQP4-M23 extracellular loop mutants including two mutations in loop A (serial AA substitution, insertion of a myc-tag), two in loop C (N153Q, insertion of a myc-tag), and two in loop E (H230G, insertion of a myc-tag). Fourty-seven baseline and 49 follow-up serum samples and six paired cerebrospinal fluid (CSF) baseline samples of 47 AQP4-IgG-positive Austrian NMOSD patients were then tested for their binding capability to AQP4-M1 and AQP4-M23 isoforms and these six extracellular loop mutants.

**Results:**

Overall, we could identify two broad patterns of antibody recognition based on differential sensitivity to mutations in extracellular loop A. Pattern A was characterized by reduced binding to the two mutations in loop A, whereas pattern B had only partial or no reduced binding to these mutations. These two patterns were not associated with significant differences in demographic and clinical parameters or serum titers in this retrospective study. Interestingly, we found a change of AQP4-IgG epitope recognition pattern in seven of 20 NMOSD patients with available follow-up samples. Moreover, we found different binding patterns in five of six paired CSF versus serum samples, with a predominance of pattern A in CSF.

**Conclusions:**

Our study demonstrates that AQP4-IgG in sera of NMOSD patients show distinct patterns of antibody recognition. The clinical and diagnostic relevance of these findings have to be addressed in prospective studies.

**Electronic supplementary material:**

The online version of this article (doi:10.1186/s12974-016-0642-3) contains supplementary material, which is available to authorized users.

## Background

Neuromyelitis optica (NMO) is a rare but devastating autoimmune and demyelinating disease of the central nervous system (CNS), usually characterized by optic neuritis (ON) and/or longitudinally extensive transverse myelitis (LETM) [1]. The discovery of a highly specific autoantibody against the aquaporin-4 (AQP4) water channel (AQP4-IgG) unified a spectrum of NMO-related disorders and distinguished them from multiple sclerosis (MS) [2]. In 2015, the International Panel for NMO Diagnosis (IPND) revised the NMO diagnostic criteria and defines the new nomenclature for the unifying term NMO spectrum disorders (NMOSD) [3]. It was shown that human AQP4-IgG enters the CNS through a leaky blood-brain barrier (BBB) resulting from inflammation and leads to a cascade of complement activation and primary astrocytopathy followed by recruitment of inflammatory cells, finally leading to oligodendrocyte injury and demyelination [4–7]. The specific target antigen is the AQP4 water channel located on astrocytic end-feet processes, facing the blood-brain and brain-CSF interfaces as well as on ependymal cells lining the ventricles and on sensory organs such as retinal Müller cells [8–10]. It consists of six transmembrane helical domains and therefore forms three extracellular loops A, C, and E in which defined amino acids (AA) were already proven to be critical for AQP4-IgG binding [11–15]. However, the clinical relevance of these findings is still unclear. There are two AQP4 isoforms, a long M1 isoform with translation initiation at Met-1 and a short M23 isoform with translation initiation at Met-23 [16]. The M23 isoform aggregates in the membrane to orthogonal arrays of particles (OAPs) and was already proven to have a higher AQP4-IgG binding specificity than the M1 isoform [12, 15, 17–20]. Moreover, it has been shown that the interaction between AQP4-IgG and OAPs induces pathogenic mechanisms such as complement-dependent cytotoxicity (CDC) and antibody-dependent cellular cytotoxicity (ADCC) [21–24]. Binding of AQP4-IgG to AQP4 OAPs was shown to greatly increase CDC involving C1q binding to the IgG1 Fc region [25]. However, AQP4-IgG does not bind to other OAP-forming aquaporins such as AQP0 and AQPcic [12]. Therefore, it seems to be a combination of specific AQP4 AA sequences leading to unique interactions of the extracellular loops that evoke high binding of AQP4-IgG and subsequent activation of an inflammatory immune cascade. Early diagnosis and discrimination from MS is very important since NMOSD cause severe neurologic impairment and requires different and subsequent treatment. Therefore, the demand for AQP4-IgG testing increased over the last decade and different assays for the detection of AQP4-IgG were developed including cell-based assays using live or fixed cells, flow cytometry, immunohistochemistry, and ELISA [26–30]. The aim of this study was to establish a sensitive and specific cell-based flow cytometry assay for the detection and quantification of serum AQP4-IgG antibodies. Moreover, we aimed to characterize specific antibody binding patterns to AQP4 epitopes in AQP4-IgG-positive serum samples of NMOSD patients based on recent findings by Owens et al. who identified specific amino acid residues on AQP4 extracellular loops A, C, and E as important epitopes for binding of human recombinant monoclonal AQP4 specific antibodies and defined restricted binding patterns [14]. For the evaluation of specific patterns of antigen recognition, we tested 47 AQP4-IgG positive serum samples and 49 follow-up samples for their capability to bind AQP4-M1 and AQP4-M23 wild-type isoforms and six AQP4-M23 extracellular loop mutants. Additionally, we analyzed six paired CSF samples that were collected at baseline.

## Methods

### Patients and serum samples

Serum samples for this retrospective case-control study were collected in the Clinical Departments of Neurology (Medical Universities of Innsbruck, Graz, and Vienna; Austria) between 2005 and 2016 and stored at −20 °C until use. Serum samples were obtained from 47 AQP4-IgG-positive NMOSD patients (40 females and 7 males, mean age 49 years, 95 % confidence interval (CI) 44–54 years) diagnosed according to the international consensus diagnostic criteria 2015 [3]. The AQP4-IgG serostatus of these patients was defined using a life immunofluorescence cell-based assay for AQP4-M23 antibodies [18]. This assay was validated to be highly accurate in the recently published European multicenter study of AQP4-IgG assays in NMOSD [20]. NMOSD patients are part of an ongoing national epidemiological study on NMOSD in Austria, and their clinical data were described in detail before [18, 31]. We included 22 patients with clinically definite NMO (20 females, 2 males, mean age 50 years, 95 % CI 43–57 years), 14 patients with isolated LETM (11 females and 3 males, mean age 57 years, 95 % CI 48–66 years), and 11 patients with isolated ON (9 females and 2 males, mean age 37 years, 95 % CI 26–49 years). Fourteen patients had an acute relapse (30 %). In addition to these 47 baseline samples, we also analyzed 49 follow-up samples (mean follow-up 3.1 years, 95 % CI 1.9–4.4 years) from 20 patients (11 NMO, 4 LETM, 5 ON; 18 females, 2 males) and 6 paired baseline CSF samples from 5 NMO and 1 ON patient (5 females and 1 male).

One hundred ninety-seven serum samples served as controls for the establishment of the AQP4-IgG flow cytometry assay. We included 68 patients with MS (44 female and 24 male, mean age 42 years, 95 % CI 40–44 years), 40 patients with other neurological diseases (OND; 14 female and 26 male, mean age 54 years, 95 % CI 48–60 years), and 89 healthy controls (HC; 39 female and 50 male, median age 50 years, range 48–53 years). Most of the patients have previously been included in other studies on AQP4 autoantibodies [4, 18].

This study was approved by the ethical committee of the Medical University of Innsbruck (study number AM3041A), Medical University of Graz and Medical University of Vienna. All patients, controls, or their legal representatives gave written informed consent to the patient or proband consent form.

### Mutagenesis of M23 AQP4

**Fig. 1:**
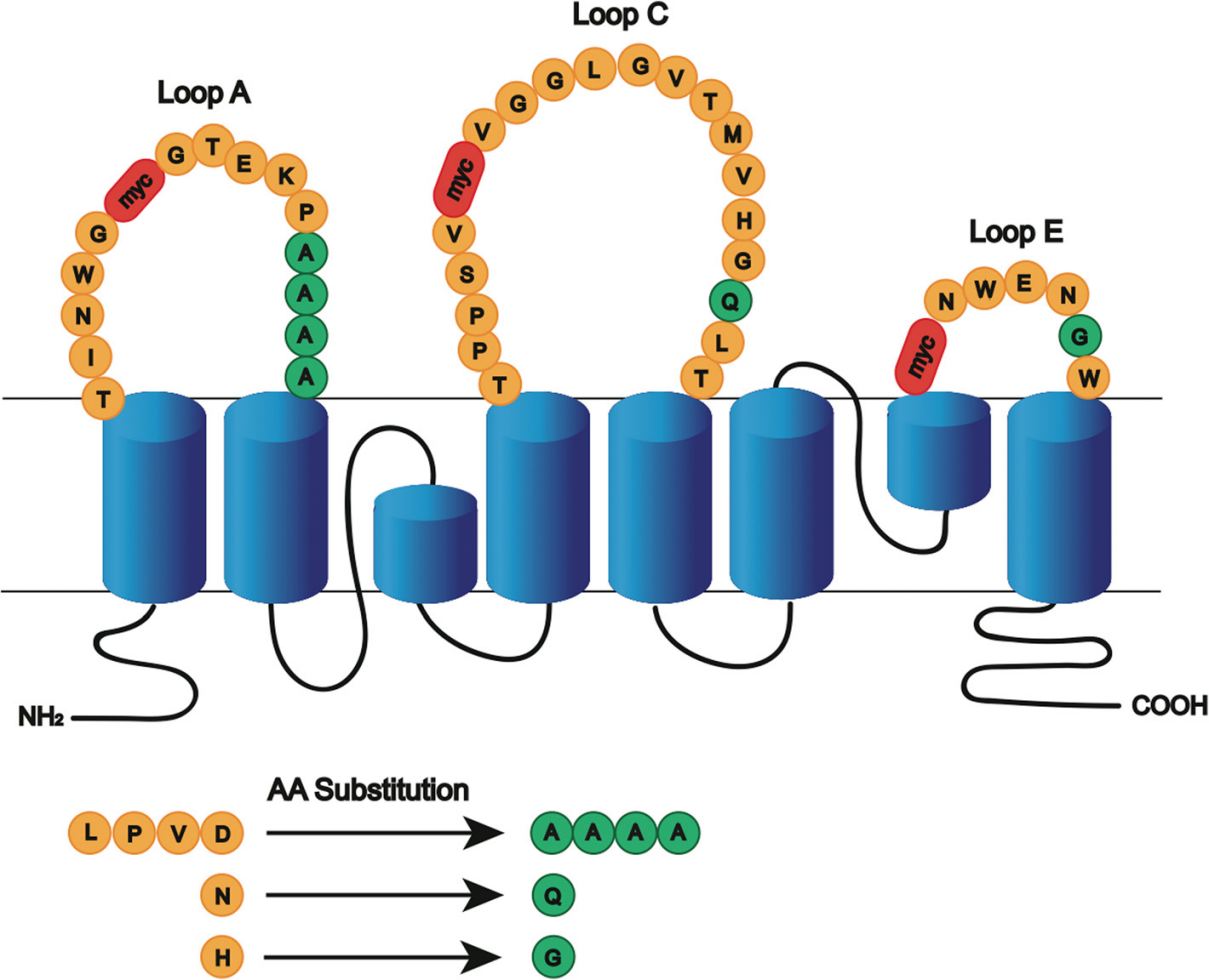
Schematic model of mutations performed in AQP4 extracellular loops. AQP4 schematic showing the positions of mutations introduced into the extracellular domains. Original amino acids (*orange*), amino acid substitutions (*green*), and the inserted myc sequence (*red*)

For the generation of all AQP4 mutants the vector construct pcDNA6.2 (M23) AQP4-EmGFP was used [18]. Both myc-tags and a 4-amino acid serial substitution as well as point mutations were introduced into the AQP4 extracellular loops using Quickchange II Site-Directed Mutagenesis Kit (Agilent, #200521). Vector constructs and primers were designed using Vector NTI Advance Software (Invitrogen) according to the instructions for the Quickchange II Site-Directed Mutagenesis Kit. All primers were made by custom oligonucleotide synthesis from Microsynth and are shown in Additional file 1. All constructs were fully sequenced (Microsynth). All mutations are shown in Table 1 and Fig. 1.

**Table 1:** Extracellular loop mutations

Loops	Name	WT sequence	Mutated sequence
A	AQP4-delA3	^66^LPVD^69^	^66^AAAA^69^
A	AQP4-mycA	^59^WGGT^62^	^59^WG-myc-GT^62^
C	AQP4-N153Q	^150^VHGNLT^155^	^150^VHGQLT^155^
C	AQP4-mycC	^140^SVVG^143^	^140^SV-myc-VG^143^
E	AQP4-H230G	^228^ENHW^231^	^228^ENGW^231^
E	AQP4-mycE	^226^NWEN^229^	^226^myc-NWEN^229^

#### Myc-tag

To change the conformation of the extracellular loops A, C, and E, a 10-amino acid long myc sequence (H-Glu-Gln-Lys-Leu-Ile-Ser-Glu-Glu-Asp-Leu-OH) was introduced at position G^60^ of extracellular loop A (mycA), at position V^141^ of extracellular loop C (mycC) and at position G^225^ of extracellular loop E (mycE) based on previous studies [14, 32].

#### Single point and serial substitutions

Extracellular loops A, C, and E of AQP4-M23 were mutated by site-directed mutagenesis as described before [14]. A serial substitution in the extracellular loop A replaced the amino acid sequence ^66^LPVD^69^ by four alanine residues (delA3). Furthermore, a single point mutation in extracellular loop C replaced asparagine with glutamine (N153Q) and in the extracellular loop E, histidine was replaced by glycine (H230G). Mutations were chosen based on previous studies [13, 14].

### AQP4-IgG cell-based flow cytometry assay

Human embryonic kidney cells HEK-293A (ATCC, LGC Standards GmbH) were seeded in 3 ml of DMEM medium supplemented with 4.5 g/l glucose (Invitrogen Life Technologies, #41965-039), 1 % non essential amino acid (NEAA, Invitrogen #11140-035), 200 mM L-glutamine (Invitrogen #11140-035) and 10 % fetal calf serum (FCS, Invitrogen #10270) in 6-well plates (TPP Switzerland, #92006) at a density of 4 × 10^5^ cells per well. Approximately 24 h later, cells were transiently transfected with the plasmids encoding AQP4-M1, AQP4-M23 [18], or AQP4-M23 mutations (delA3, N153Q, H230G, mycA, mycC, mycE), respectively. All resulting proteins are C-terminally fused to EmGFP. Seventy-two hours post transfection the cells were harvested. Therefore, the medium was removed and cells washed with 3 ml PBS (Sigma, #P3813). Cell layer was detached using 150 μl of 0.05 % trypsin-EDTA (Invitrogen, #25300-054). Trypsinization was stopped by adding 1 ml medium to the cells, and the suspension was transferred to a 50-ml falcon tube and centrifuged at 500×*g* for 5 min at room temperature (RT). After removing the supernatant, the cell pellet was resuspended in 10 ml FACS buffer (PBS + 10 % FCS + 1 mM EDTA) supplemented with FcR Blocking Reagent (Milteny, #130-059-901) and blocked for 1 h by shaking carefully at 4 °C. Meanwhile, serum dilutions were prepared. Each serum sample was diluted 1:10 in 100 μl FACS buffer. Serum was plated on a round-bottom 96-well plate (TPP Switzerland, #92096), 100 μl of serum dilution per well, and duplicates were used for all samples. After blocking, cells were centrifuged (500×*g*, 5 min) and the cell pellet was resuspended in FACS buffer. Approximately 150,000–200,000 cells in 100 μl FACS buffer were added to the serum dilutions (reaching the final serum dilution of 1:20) in each well. Cell/serum mixture was in incubated for 30 min by shaking carefully at 4 °C. Subsequently, cells were washed with FACS buffer by centrifugation for 5 min at 500 x g and decanting the supernatant carefully. Cells were incubated for 30 min with secondary antibody goat anti-human IgG, Fc (Dianova, #109-135-098) 1:100 dilution by shaking at 4 °C. Finally, cells were fixed in 100 μl FACS buffer supplemented with 10 μl CellFIX (BD Biosciences, #340181). AQP4-IgG binding was determined by using the BD Accuri^Tm^ C6 flow cytometer and the BD Accuri^Tm^ C6 software (BD Biosciences). Precision was determined by running all samples in duplicates in order to calculate the intra-assay variability. Samples we replicated in triplicates if the coefficient of variation was higher than 15 %. AQP4 and AQP4-mutant expressing cells were detected in the green FL-1 channel and the antibody binding was measured in the red FL-4 channel to avoid spectral overlapping. A minimum of 10,000 EmGFP^pos^ cells were acquired for each sample. For binding analysis gates were set on the cells with an emGFP expression level of FL-1 10^4^ and 10^5^ for AQP4^pos^ population as recently described for a comparable FACS autoantibody assay [33]. The AQP4^neg^ population was gated on FL-1 < 10^4^ (Additional file 2). The median fluorescence intensity (MFI) in the FL-4 channel was obtained by gating on the AQP4^pos^ and AQP4^neg^ populations. The binding ratio was calculated as MFI(AQP4^pos^)/MFI(AQP4^neg^). Cells transfected with the mutants and with AQP4-M23 WT were always measured together in the same experiment to determine the binding percentage as percent binding = $\frac{\mathrm{MFI}\left(\mathrm{mutant}\ \mathrm{A}\mathrm{Q}\mathrm{P}4\mathrm{p}\mathrm{o}\mathrm{s}\right)-\mathrm{M}\mathrm{F}\mathrm{I}\left(\mathrm{mutant}\ \mathrm{A}\mathrm{Q}\mathrm{P}4\mathrm{n}\mathrm{e}\mathrm{g}\right)}{\mathrm{MFI}\left(\mathrm{W}\mathrm{T}\ \mathrm{A}\mathrm{Q}\mathrm{P}4\mathrm{p}\mathrm{o}\mathrm{s}\right)-\mathrm{M}\mathrm{F}\mathrm{I}\left(\mathrm{W}\mathrm{T}\ \mathrm{A}\mathrm{Q}\mathrm{P}4\mathrm{n}\mathrm{e}\mathrm{g}\right)} \times 100\%$.

### Recombinant AQP4-specific monoclonal antibodies

Recombinant monoclonal AQP4-specific antibodies (AQP4 rAbs (kindly provided by Prof. Jeffrey L. Bennett from the University of Colorado, Denver, CO) were generated from clonally expanded cerebrospinal fluid (CSF) plasma blasts as described previously [5]. For binding specificities of human AQP4 rAbs to AQP4-M1 and AQP4-M23 isoforms cell-based flow cytometry assay was performed as described before. Briefly, 1 μg of each AQP4 rAb was diluted in 50 μl FACS buffer and plated on a round-bottom 96-well plate. To the rAb dilutions (reaching a final dilution of 1 μg/100 μl (10 μg/ml)) in each well, 150,000–200,000 cells in 50 μl FACS buffer were added. All samples were run in duplicates. For determination of binding, constants of AQP4 rAbs to AQP4-M23 Abs were diluted from 100 to 0.8 μg/ml and measured by cell-based flow cytometry assay described before.

### Blue native gel electrophoresis

All AQP4 mutant constructs were studied for OAP assembly by using blue native gel electrophoresis (BN-PAGE). Therefore, HEK-293A cells were transfected as described before. Cells were lysed in 1X NativePage^TM^ Sample Buffer (Invitrogen, # BN2003) supplemented with 500 mM 6-aminohexanoic acid, 1 % Triton X-100 and protease inhibitor cocktail, incubated for 30 min on ice and centrifuged at 22,000×*g* for 30 min. Supernatants were supplemented with NativePage^TM^ 5 % G-250 (Invitrogen, #BN2004) and loaded to NativePAGE™ Novex™ 3-12 % Bis-Tris Protein Gels (Invitrogen, # BN1001). The running buffers were prepared according to the manufacturer’s protocol. Proteins were blotted onto polyvinylidene difluoride (PVDF) membrane using NuPage Transfer Buffer (Invitrogen, #NP0006). For immunoblot analysis, proteins were fixed for 15 min in 8 % acetic acid and membranes were blocked in 1 % Amersham ECL Prime Blocking Reagent (GE Healthcare, # RPN418) diluted in 0.1 % PBS-Tween. Primary rabbit anti-AQP4 antibody (Sigma, # A5971) was incubated at 4 °C overnight. Secondary antibody Amersham ECL Rabbit IgG (GE Healthcare, # Na934) was incubated at RT for 1 h. Labeled proteins were detected using the WesternBright ECL (Biozym, # 541004) and visualized on the Fusion FX7 Vilber Lourmat imaging system.

### Statistical analysis

Statistical analysis was done using IBM SPSS software (release 22.0, IBM) or GraphPad Prism 6 (GraphPad). Between-group comparisons were performed with ANOVA or Kruskal-Wallis test, *T* test or Mann-Whitney *U* test, Fisher’s exact test, and chi-square test. Correlation of parameters was analyzed with Spearman’s non-parametric correlation. Receiver operating characteristic (ROC) curve analysis was used to determine cutoff FACS binding ratios and immunological parameters determining antibody binding patterns. The effect of demographic and clinical parameters on binding patterns was analyzed using binary logistic regression analysis. Heatmaps were drawn using GENE-E matrix visualization and analysis software (http://www.broadinstitute.org/cancer/software/GENE-E/index.html). Statistical significance was defined as two-sided *p* value <0.05 and Bonferroni corrections were applied for multiple comparisons when appropriate.

## Results

### Establishment of a flow cytometry based detection method for AQP4-IgG autoantibodies

**Fig. 2:**
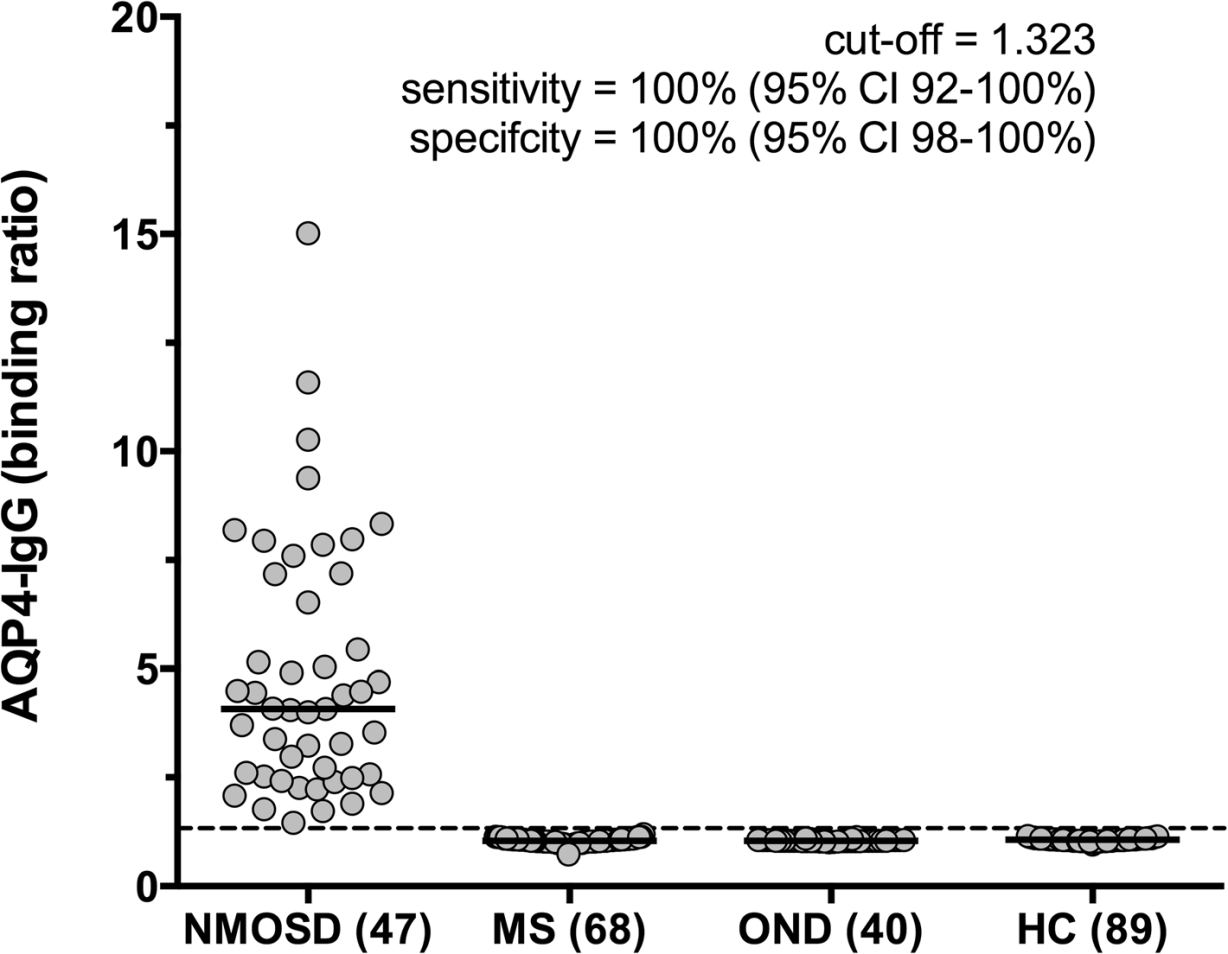
AQP4-M23 binding ratios in seropositive NMOSD patients and seronegative control groups. AQP4-IgGs were exclusively detected in serum samples of patients with NMOSD, but not in MS patients, patients with OND or HC. The cutoff value of 1.323 is indicated by a *dashed horizontal line*. Medians are indicated by *horizontal bars*. Binding ratios were compared by using a non-parametric test (Kruskal-Wallis Test) revealing *p* < 0.0001. *NMOSD* neuromyelitis optica spectrum disorders, *MS* multiple sclerosis, *OND* other neurological diseases, *HC* healthy controls

We established a cell-based flow cytometry assay as detection method for AQP4-antibodies and screened 47 AQP4-IgG positive baseline serum samples of 47 NMOSD patients and 197 AQP4-IgG negative control serum samples for their binding ratio against the AQP4-M23 isoform expressed on HEK-293A cells (Fig. 2). HEK-293A cells with the fluorescence intensity of 10^4^–10^5^ were gated to determine the binding ratio to the transfected cell population and to ensure a high and comparable expression level between the transfectants (Additional file 2). A cutoff value (1.323) was calculated by the binding ratio of our seronegative control groups using ROC analysis. Serum positivity of AQP4 autoantibodies was confirmed in all NMOSD samples whereas none of the controls was seropositive, yielding an assay sensitivity and specificity of 100 % (95 % CI 92–100 % and 95 % CI 98–100 %, respectively).

Moreover, we validated our AQP4-IgG assay using human monoclonal antibodies derived from clonally expanded CSF plasmablasts of AQP4-IgG positive NMOSD patients [17]. Additional file 3A shows binding curves of five recombinant monoclonal antibodies tested for their binding affinities to the AQP4-M23 isoform expressed in HEK-293A cells by flow cytometry.

### Heterogeneous AQP4-IgG binding to AQP4 isoforms and extracellular loop mutants in NMOSD serum samples

As previous studies showed that AQP4-IgGs recognize distinct conformational epitopes on AQP4 we generated six extracellular loop mutants. The serial AA substitution in loop A (delA3) and a single AA substitution in loop C (N153Q) and loop E (H230G) were already tested for their impact on binding of monoclonal rAbs described previously [14]. However, antibody recognition studies of heavily manipulated AQP4 loops by insertion of the myc epitope have to our knowledge not been reported.

As seen in Table 2, we found a reduced antibody binding capability to all mutations in 47 AQP4-IgG positive baseline serum samples. The strongest impact on antibody binding was observed in the mycE mutation with a binding capability of 5.4 % (95 % CI 3.7–7.1) compared to AQP4-M23, followed by the mycC mutation with 13.1 % (95 % CI 9.3–16.9) and the mycA mutation with 38.1 % (95 % CI 29.0–47.4). This reduction of antibody binding is also seen for AQP4-IgG binding ratios (Table 2). To confirm surface localization of the myc epitope, live cell immunofluorescence stainings were performed using an anti-myc antibody. Myc epitopes of loop A and loop C showed typical surface stainings, whereas loop E could not be detected on the surface. However, after fixation intracellular expression was confirmed.

**Table 2:** Antibody binding of 47 AQP4-IgG-positive baseline serum samples to AQP4-M23, AQP4-M1 and AQP4-M23 mutants

	AQP4-IgG binding ratio (mean, 95 % CI)	AQP4-IgG binding in % of AQP4-M23 (mean, 95 % CI)
AQP4-M23 isoform	4.84 (3.98–5.70)	Reference (100 %)
AQP4-M1 isoform	1.48 (1.11–1.86)	25.83 (20.19–31.47)
AQP4-mycA (loop A)	2.74 (2.17–3.31)	38.15 (28.95–47.35)
AQP4-delA3 (loop A)	2.85 (2.26–3.44)	40.91 (31.99–49.82)
AQP4-mycC (loop C)	1.49 (1.38–1.60)	13.12 (9.34–16.90)
AQP4-N153Q (loop C)	3.88 (3.26–4.50)	67.35 (56.88–77.81)
AQP4-mycE (loop E)	1.17 (1.12–1.23)	5.39 (3.69–7.08)
AQP4-H230G (loop E)	2.69 (2.12–3.25)	36.28 (29.62–42.95)

In contrast to loops containing myc epitopes, single point mutations showed less influence on antibody binding in the case of H230G (36.3 %, 95 % CI 29.6–43.0) and N153Q (67.3 %, 95 % CI 56.9–77.8). However, in loop A the delA3 substitution presented with a mean binding of 40.9 % (95 % CI 32.0–49.8), which is comparable to the result of mycA. As seen in Table 2, both the binding ratio and the range of percent binding varied considerably in every investigated mutation or the AQP4-M1 isoform.

Finally, we analyzed the role of OAP formation in the observed reduced antibody binding. Whereas the three point mutations and the myc-tag in loop A did not affect OAP assembly, introduced myc-tags in loops C and E caused OAP disruption as distinct tetramers and higher order arrays are absent when visualized by BN-PAGE (Additional file 4).

### Mutagenesis of AQP4 extracellular loop A identifies two broad patterns of serum AQP4-IgG recognition

**Fig. 3:**
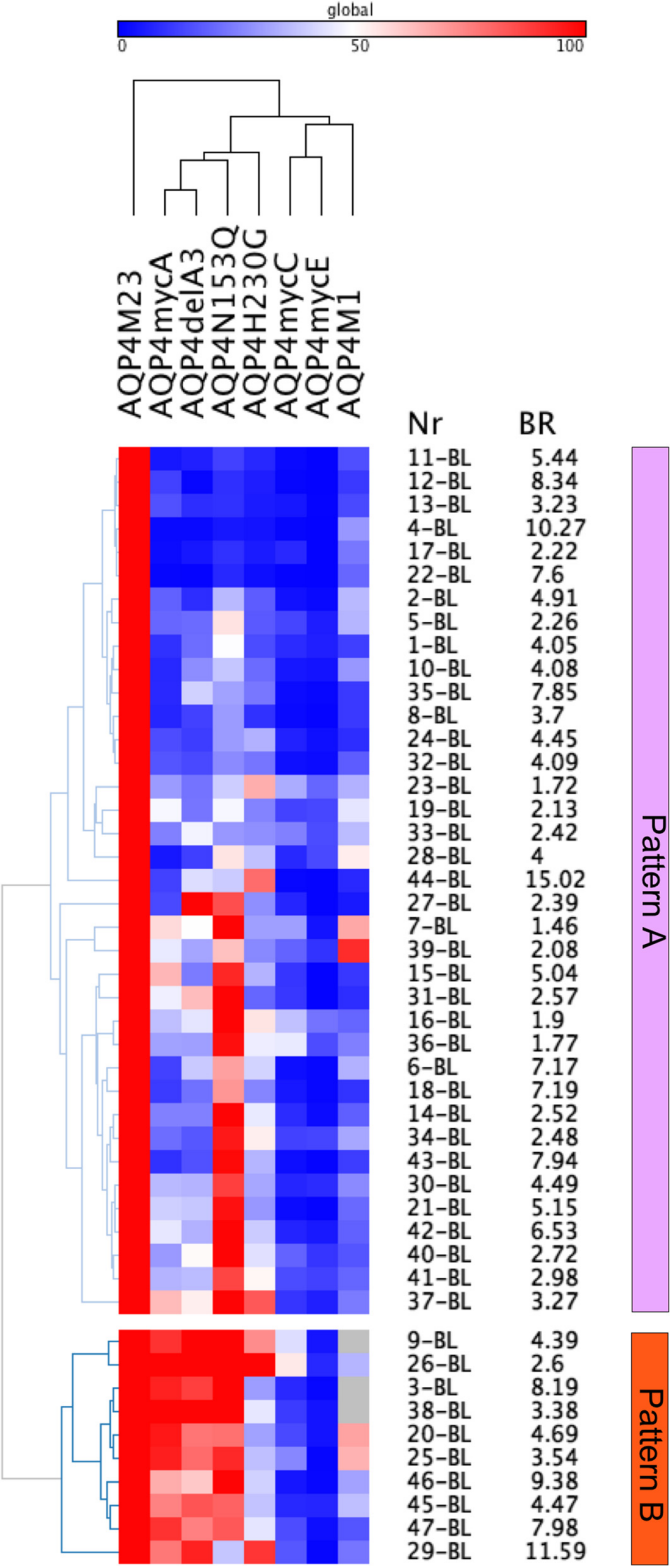
Heatmap of serum AQP4-antibody levels against AQP4-M23, AQP4-M1 and AQP4-M23 mutants (*columns*) at baseline. *Rows* are individual samples with patient IDs (Nr) and flow cytometry AQP4-M23 binding ratios (BR) shown at the right side. Data are shown as percent binding of AQP4-M23. Values range from *blue* (0 %) to *white* (50 %) to *red* (100 %). Columns were clustered according to their Pearson’s correlation coefficients, and rows were clustered according to their Eucledian distance (both average linkage). Two major antibody binding patterns were identified, a loop A-dependent pattern A (*upper panel*) and an independent pattern B. The heatmap was generated using GENE-E matrix visualization and analysis software (http://www.broadinstitute.org/cancer/software/GENE-E/index.html)

Consistent with a recent publication on AQP4-specific rAbs recognizing restricted binding patterns [14], we also identified two broad binding patterns for serum antibody recognition comprising a mainly loop A-dependent pattern A and a loop A-independent pattern B using the unbiased approach of hierarchical clustering (Fig. 3 and Table 3). We also tested five monoclonal rAbs for their binding capability to both loop A mutations and could confirm the results by Owens et al. who identified rAbs ON09-3 #33 and ON07-5 #58 as loop A-dependent (Additional file 3B) [14].

**Table 3:** Immunological correlates of AQP4-IgG binding patterns

	Loop A-dependent pattern A (*n* = 37)	Loop A-independent pattern B (*n* = 10)	Area under the curve (95 % CI)	*p* value*
AQP-M23 binding	4.52 (3.57–5.48)	6.02 (3.86–8.19)	0.50 (0.25–0.75)	0.999
AQP4-M1 binding	1.42 (1.01–1.83)	1.78 (0.59–2.97)	0.56 (0.33–0.78)	0.999
AQP4-M1 (% M23)	25.1 (19.3–31.1)	28.1 (10.0–31.5)	0.78 (0.59–0.97)	0.405
AQP4-mycA binding	1.95 (1.69–2.21)	5.66 (4.12–7.19)	0.99 (0.96–1.00)	<0.001
AQP4-mycA (% M23)	24.5 (18.5–30.52)	88.6 (80.4–96.8)	1.00 (1.00)	<0.001
AQP4-delA3 binding	2.15 (1.75–2.56)	5.42 (3.77–7.07)	0.96 (0.90–1.00)	<0.001
AQP4-delA3 (% M23)	28.7 (21.9–35.5)	86.2 (77.0–95.4)	0.97 (0.92–1.00)	<0.001
AQP4-mycC binding	1.36 (1.32–1.41)	1.49 (1.38–1.60)	0.88 (0.72–1.00)	0.045
AQP4-mycC (% M23)	11.1 (7.4–14.8)	20.6 (8.7–32.5)	0.72 (0.52–0. 93)	0.999
AQP4-N153Q binding	3.47 (2.86–4.07)	5.42 (3.63–7.20)	0.75 (0.58–0.92)	0.750
AQP4-N153Q (% M23)	61.3 (49.1–73.5)	89.6 (74.2–100)	0.72 (0.55–0.89)	0.999
AQP4-mycE binding	1.14 (1.11–1.17)	1.29 (1.05–1.53)	0.63 (0.33–0.92)	0.999
AQP4-mycE (% M23)	5.8 (3.7–7.9)	3.7 (2.0–5.5)	0.54 (0.36–0.71)	0.999
AQP4-H230G binding	2.35 (1.80–2.89)	3.94 (2.22–5.67)	0.71 (0.52–0.91)	0.999
AQP4-H230G (% M23)	31.3 (25.0–38.2)	53.6 (35.7–71.4)	0.74 (0.59–0.90)	0.870

AQP4-M23, AQP4-M1, and extracellular loop A, C, and E mutations were expressed in HEK-293A cells and assayed to categorize pattern A and pattern B serum samples as indicated in Fig. 3. Data are shown as means with 95 % confidence interval. *Binding* binding ratios

*The area under the curve was analyzed by receiver operator curve (ROC) analysis and *p* values were corrected for 15 comparisons

Thirty-seven (79 %) of our AQP4-IgG positive serum samples showed reduced binding to both loop A mutations (Fig. 3 and Table 3) confirming that most of our NMOSD sera required the intact extracellular loop A sequence. Using ROC analysis, we determined that pattern A AQP4-IgG positive samples were characterized by reduced binding to the delA3 mutant (cutoff <58 %) and the mycA mutant (cutoff <66 %). Importantly, AQP4-M23 binding ratios did not influence binding patterns, whereas reduced binding ratios for the delA3 mutant (cutoff <3.4) and the mycA mutant (cutoff <3.7) were significantly associated with pattern A. Moreover, we were able to verify these binding patterns by analyzing the absolute loss of binding (binding ratios) of AQP4 isoforms and mutants (Additional file 5) and by using 49 follow-up samples as a validation cohort (Additional file 6).

Both patterns were recognized by AQP4-IgG autoantibodies in the serum of patients with all three different clinical NMOSD entities: NMO, LETM, and ON. In this small cohort of NMOSD patients, the loop A-independent pattern B was more often found in patients with clinically definite NMO (8/10 versus 14/37, Table 4). Furthermore, pattern B was more often found in patients suffering from an acute relapse. But neither these nor other differences regarding sex, age, age at disease onset, disease duration, number of relapses, disability (EDSS), or treatment reached statistical significance.

**Table 4:** Clinical and demographic characteristics of NMOSD patients according to AQP4-IgG binding pattern at baseline

	Loop A-dependent pattern A (*n* = 37)	Loop A-independent pattern B (*n* = 10)	*p* value
Females^a^	31 (84 %)	9 (90 %)	0.125
Age (years)^b^	49.3 (43.6-55.1)	49.0 (36.1-62.0)	0.764
Age at onset (years)^b^	41.1 (35.2-47.1)	42.8 (26.3-59.2)	0.722
Disease duration (years)^b^	8.6 (6.4-10.9)	6.8 (0–13.9)	0.662
Acute relapse^a^	8 (22 %)	6 (60 %)	0.550
EDSS^c^	3 (0.5-8.5)	5 (2–7)	0.196
Relapses^c^	3 (1–19)	3 (1–11)	0.109
Diagnosis^a^			
NMO	14 (38 %)	8 (80 %)	0.455
LETM	14 (38 %)	0 (0 %)	
ON	9 (24 %)	2 (20 %)	
Treatment^d^			
No treatment	4	2	0.367
Corticosteroids	5	1	
Azathioprine	10	4	
Rituximab	14	1	
Other treatments	4	2	

AQP4-M23, AQP4-M1 and extracellular loop A, C, and E mutations were expressed in HEK-293A cells and assayed to categorize pattern A and pattern B serum samples as indicated in Fig. 3. Other treatments = interferon-ß (1), cyclophosphamide (1), mitoxanthrone (1), intravenous immunoglobulins (2) and plasma exchange (1). The effect of demographic and clinical parameters on binding patterns was statistically analyzed using binary logistic regression analysis

^a^Number of cases (%)

^b^Mean (95 % confidence interval)

^c^Median (range)

^d^Number of cases

**Fig. 4:**
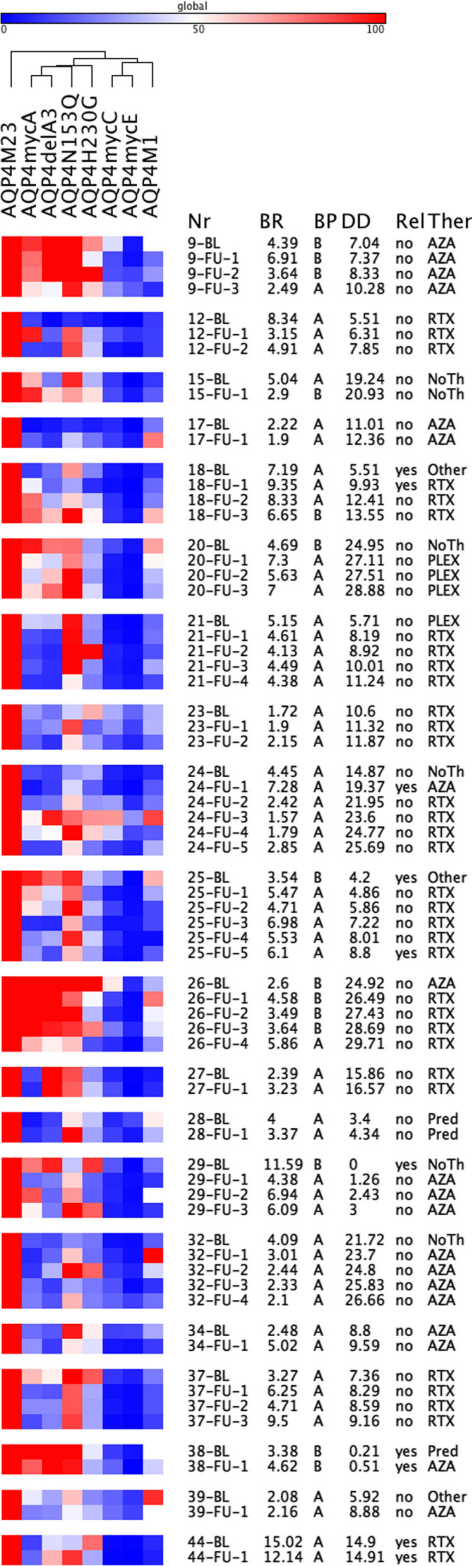
Heatmap of serum AQP4-antibody levels against AQP4-M23, AQP4-M1 and AQP4-M23 mutants (*columns*) in baseline and follow-up samples of 20 NMOSD patients with follow-up samples. *Rows* are individual samples with patient IDs (Nr), flow cytometry AQP4-M23 binding ratios (BR), AQP4-IgG binding patterns (BP), disease duration in years (DD), presence of acute relapses (Rel), and therapies (Ther) shown at the *right side*. Data are shown as percent binding of AQP4-M23. Values range from *blue* (0 %) to *white* (50 %) to *red* (100 %). Columns were clustered according to their Pearson’s correlation coefficients. Two major antibody binding patterns were identified, a loop A-dependent pattern A and an independent pattern B. The heatmap was generated using GENE-E matrix visualization and analysis software (http://www.broadinstitute.org/cancer/software/GENE-E/index.html). *AZA* azathioprine, *NoTh* no therapy, *other* other immunsuppressive therapies, *PLEX* plasma exchange, Pred _ corticosteroids, *RTX* rituximab

In addition to the 47 baseline samples, we have also analyzed 49 follow-up samples from 20 patients. Thirteen patients (12 females, 1 male; 6 NMO, 4 LETM, 3 ON) showed stable antibody binding pattern (12 with pattern A and 1 with pattern B), and seven patients (6 females, 1 male; 5 NMO, 2 ON) showed a change in antibody binding patterns (Fig. 4). Two patients showed a change from pattern A → B and five patients showed a change from pattern B → A. The recognition pattern changes of all follow-up samples and their relation to absolute antibody binding values, disease duration, presence of acute relapse, and therapies are shown in Fig. 4. There was no obvious association with the presence of relapses (5/29 in the group with no changes and 5/29 in the group with changes), specific treatments, or treatment changes. However, the clinical relevance of these observations needs to be analyzed in a larger prospective study.

### Different patterns of AQP4-IgG recognition in CSF and serum samples

**Fig. 5:**
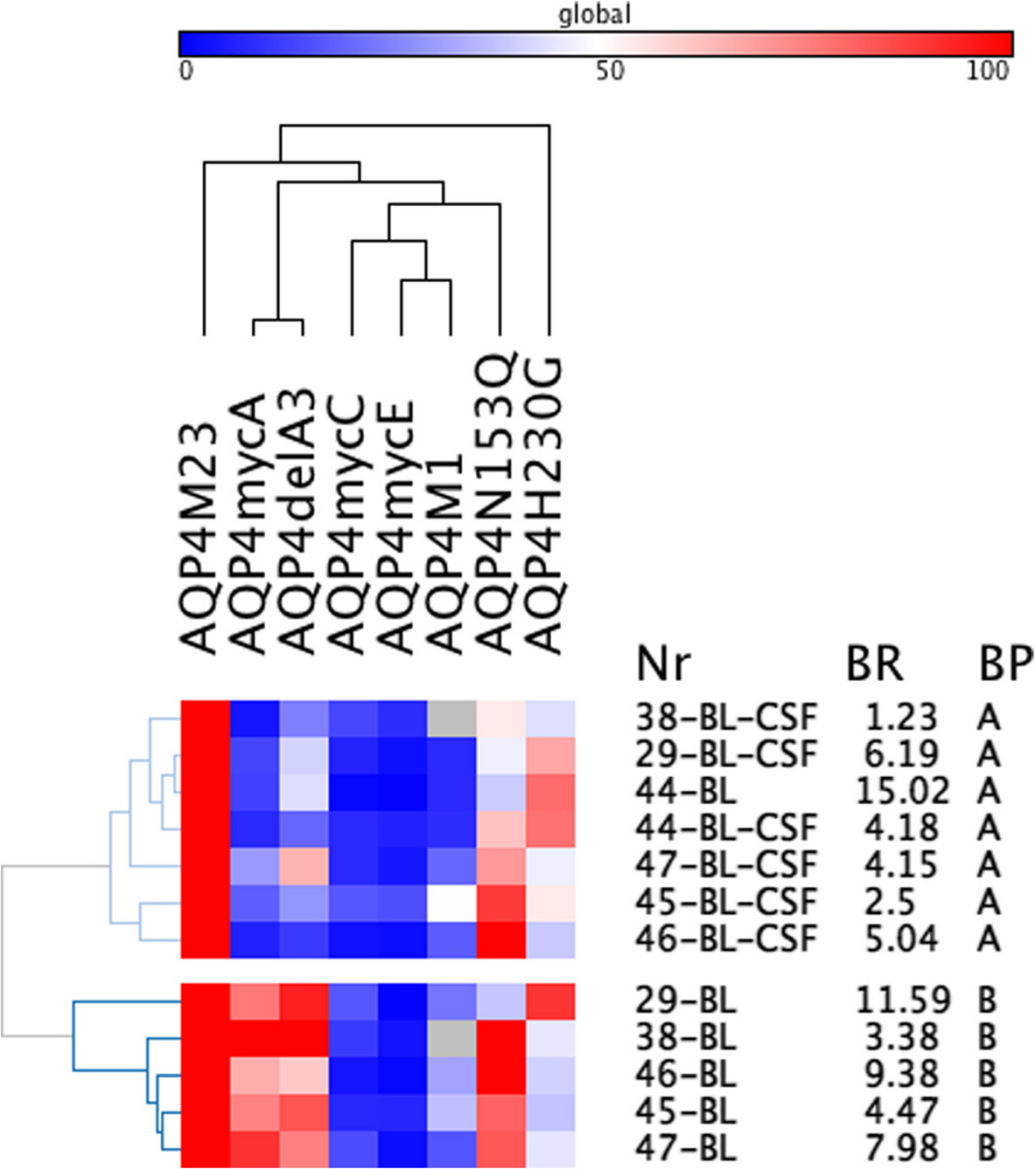
Heatmap of CSF and serum AQP4-antibody levels against AQP4-M23, AQP4-M1, and AQP4-M23 mutants (*columns*) in baseline samples of 6 NMOSD patients with paired CSF and serum samples. *Rows* are individual samples with patient IDs (Nr), flow cytometry AQP4-M23 binding ratios (BR), and AQP4-IgG binding patterns (BP) shown at the right side. Data are shown as percent binding of AQP4-M23. Values range from *blue* (0 %) to *white* (50 %) to *red* (100 %). Columns were clustered according to their Pearson’s correlation coefficients and rows were cluster according to their Eucledian distance (both average linkage). Two major antibody binding patterns were identified, a loop A-dependent pattern A and an independent pattern B. Five of the six patients had different antibody recognition patterns in CSF and serum. The heatmap was generated using GENE-E matrix visualization and analysis software (http://www.broadinstitute.org/cancer/software/GENE-E/index.html)

Finally, we had the chance to analyze six paired CSF and serum samples taken at baseline. Surprisingly, we found different binding patterns in the CSF versus serum samples that were taken at the same time point in five of six patients (Fig. 5). Whereas all CSF AQP4-IgG antibodies had a loop A-dependent pattern A, five of six serum antibodies had a loop A-independent pattern B. The clinical, CSF, and demographic characteristics of these six NMOSD patients are shown in Table 5.

**Table 5:** Clinical, CSF, and demographic characteristics of NMOSD patients with available paired CSF and serum samples

Patient number	29	38	44	45	46	47
Sex	Female	Female	Female	Female	Female	Male
Age (y)	73.7	52.4	41.4	50.1	28.3	68.9
Age at onset (y)	73.7	52.2	26.4	49.6	25.6	68.9
Disease duration (y)	0.00	0.21	14.90	0.50	2.72	0.01
Diagnosis	NMO	ON	NMO	NMO	NMO	NMO
Acute relapse	yes	yes	yes	yes	yes	no
Number of relapses	1	1	5	2	6	1
EDSS	2.0	2.0	6.5	2.5	6.0	5.5
Treatment	NoTh	Pred	RTX	AZA	RTX	PLEX
CSF cells/μl	9	4	5	1	4	73
Albumin quotient	6.59	3.97	6.20	8.92	2.32	18.12
IgG index	0.64	0.55	0.48	0.58	1.20	0.58
CSF OCB	Negative	Negative	Negative	Negative	Positive	Negative
AQP4-IgG CSF^a^	6.19	1.23	4.18	2.50	5.04	4.15
AQP4-IgG serum^a^	11.59	3.38	15.02	4.47	9.38	7.98
AQP4-IgG quotient	0.53	0.36	0.28	0.56	0.54	0.52
AQP4-IgG pattern CSF	A	A	A	A	A	A
AQP4-IgG pattern serum	B	B	A	B	B	B

AQP4-M23, AQP4-M1 and extracellular loop A, C, and E mutations were expressed in HEK-293A cells and assayed to categorize pattern A and pattern B serum samples as indicated in Fig. 3

*AZA* azathioprine, *NoTh* no therapy, *PLEX* plasma exchange, *Pred* corticosteroids, *RTX* rituximab, *y* years, *albumin quotient* CSF albumin/serum albumin, *IgG index* albumin quotient/IgG quotient, *OCB* oligoclonal bands, *AQP4-IgG quotient* CSF/serum AQP4-IgG

^a^FACS AQP4-IgG binding ratio for AQP4-M23

## Discussion

In this study, we analyzed defined epitopes in the extracellular loops of AQP4 recognized by AQP4-IgG positive serum and CSF samples of 47 NMOSD patients. Overall, we found reduced binding to all mutations and AQP4-M1 compared to the AQP4-M23 isoform and distinguished two broad patterns of AQP4-IgG recognition, a loop A-dependent pattern A and a loop A-independent pattern B. Further, we observed reduced binding to multiple AQP4 mutants in the majority of all NMOSD patients indicating that the human AQP4 antibody response is defined by multiple epitopes. These findings are consistent with previous studies showing that serum autoantibodies bind to multiple targets including AQP4 peptides, monomers, and higher order arrays [15, 34]. A comparable study on conformational epitopes of the myelin oligodendrocyte glycoprotein (MOG) could show that the immune response can be directed against one single or multiple epitopes [33].

Introduction of a myc-tag in the extracellular loops A, C, and E reduced the recognition of AQP4 to 38.2 % (range 29.0–47.4 %), 13.1 % (range 9.3–16.9 %), and 5.4 % (range 3.7–7.1 %), respectively, indicating that elongation of the extracellular loop sequences caused serious conformational changes with great impact on antibody recognition. AA G60 in loop A was previously reported as less critical for AQP4-IgG binding and therefore selected for insertion of the myc-tag sequence [14]. As we could demonstrate by BN-PAGE, introduction of the myc-tag did not affect OAP assembly and caused minor reduction in antibody binding (38.2 %, 95 % CI 29.0–47.4; binding ratio 2.7, 95 % CI 2.2–3.3). However, the observed decrease in antibody recognition of 37 of 47 baseline serum samples indicates either the loss of specific loop A epitopes by the myc insertion or its influence on the conformation of nearby loops within the tetramer structure.

In contrast to the mycA mutation, both mycC and mycE mutations exhibited loss of OAP formation and a dramatic reduction of antibody recognition. The position for the myc-tag insertion into the extracellular loop C was based on previous studies by Crane et al. who demonstrated that position V141 did not affect AQP4 assembly into OAPs when transfected into COS-7 cells [32, 35]. In contrast to this study, we could not detect OAP formation when transfecting HEK-293A cells with the mycC mutant construct and detected major reduction in antibody recognition (13.1 %, 95 % CI 9.3–16.9; binding ration 1.5, 95 % CI 1.4–1.6). The position H230 in loop E was chosen by the study by Owens et al. who demonstrated minor impact in rAb binding [14]. However, we proved the mycE mutant to cause OAP disruption and consequently detected highly impaired AQP4-IgG binding (5.4 %, 95 % CI 3.7–7.1; binding ratio 1.2, 95 % CI 1.1–1.2).

As disruption of OAP formation in both mycC and mycE mutants severely decreases the binding capability of all serum samples, we could not find significant differences between the analyzed serum samples. The physiological role of AQP4 OAPs is still unknown but these aggregates were already proven to have a higher AQP4-IgG binding specificity [12, 17–19]. Moreover, it was shown that AQP4-IgG CDC is dependent on OAP assembly [36]. Interestingly, other OAP-forming AQP4 proteins, such as the lens AQP-0 and the insect AQP-cic, were not recognized by AQP4-IgG, suggesting that defined epitopes have key functions in antibody recognition [12, 37]. In summary, our study suggests that assembly into OAPs appears to be a key factor for binding of AQP4-IgG as our serum samples did not only show a significant reduction in binding to the non-OAP-forming AQP4-M1 isoform but also to the non-OAP-forming mycC and mycE mutant constructs.

In contrast, single point mutations N153Q (loop C) and H230G (loop E) did not disrupt OAP formation but also caused reduced binding of AQP4-IgG. However, impact on antibody binding was not as pronounced, resulting in slightly reduced binding to the N153Q mutation in loop C (67.4 %, 95 % Cl 56.9–77.8, binding ratio 3.9, 95 % CI 3.3–4.5) and the H230G mutation in loop E (36.3 %, 95 % Cl 29.6–43.0, binding ratio 2.7, 95 % CI 2.2–3.3) compared to AQP4-M23 WT (100 % reference, binding ratio 4.8, 95 % CI 4.0–5.7). Although these single mutations were shown to have a remarkable role in antibody recognition as they significantly reduced binding of all rAbs [14], we detected only minor reductions compared to the myc-tag mutants, indicating that most of the serum samples analyzed are polyclonal in nature and therefore bind to different epitopes on the AQP4 protein.

Overall, we identified two broad binding patterns for serum antibody recognition comprising a mainly loop A-dependent pattern A in 37 of 47 serum samples, based on the serial AA substitution delA3 and the mycA mutation, and a loop A-independent pattern B in 10 of 47 serum samples that showed a broader recognition pattern. This fits well to the data of Owens et al. who could also categorize rAbs into loop A-dependent and loop A-independent patterns [14]. The serial AA substitution in loop A (delA3) had a comparable impact on AQP4-IgG binding (40.9 %, range 32.0–49.8 %; binding ratio 2.9, 95 % CI 2.3–3.4) as the mycA mutation (38.2 %, range 29.0–47.4 %; binding ratio 2.7, 95 % CI 2.2–3.3). Similar findings were previously reported by Pisani et al. who detected a reduced binding in five of eleven NMO sera to a serial AA deletion at a different position in the extracellular loop A [12]. Furthermore, the delA3 include the substitution at Asp69 that have been previously reported as the most sensitive residue in the extracellular loop A as it impaired AQP4-IgG binding in 93 % of NMO patient sera [13] and produced a significant loss of binding for all loop A-dependent rAbs [14]. These defined binding patterns recognized by NMOSD patients with different disease entities (NMO, LETM, ON) did not reveal any statistically significant association between epitope recognition and disease phenotype. Analysis during the course of disease revealed a change of AQP4-IgG epitope recognition in 7/20 NMOSD patients with long-term persistence of AQP4-IgG. These patients had a change from a loop A-dependent pattern A to a loop A-independent pattern B (5 cases) or vice versa (2 cases) during their disease course. In contrast, in a previous study on patients with anti-MOG antibodies, the recognized epitopes remained constant in nine of nine analyzed cases [33]. However, we did not observe any significant association between a change in the pattern and acute relapses, a change in treatment, or a specific treatment such as B cell depletion.

Interestingly, we found different binding patterns in the CSF versus serum compartment in five out of six NMOSD patients. CSF and serum samples were taken at the same time point and analyzed for their binding capability to each mutation. We detected a loop A-dependent pattern A in all six CSF samples versus a loop A-independent pattern B in five of six serum samples suggesting a restricted intrathecal AQP4 epitope recognition. Previous findings suggested that these antibodies are mainly produced in the periphery [38, 39] but it becomes more and more evident that intrathecal AQP4 specific B cells and their corresponding plasma cells might also be a source of AQP4 specific CNS antibodies [5, 40].

Finally, our study might also help to explain the differences between sensitivities of immunoassays used for the measurement of AQP4-IgG antibodies and cell-based assays, which can detect antibodies to all AQP4 epitopes being the most sensitive assays [27, 29].

Our study has the following limitations: (1) we have analyzed only a small proportion of all potential immunodominant epitopes recognized by NMOSD patients and since OAP disrupting mutants do not make a contribution to separation into specific binding patterns, future studies should focus on mutations that do not affect OAP assembly. A systematical epitope analysis as it was performed with human rAbs [14] might identify defined amino acids and specific epitopes. (2) As this was a retrospective case-control study, we cannot make any definite conclusion on the clinical relevance of our findings. Therefore, the clinical and diagnostic relevance of our findings have to be addressed in prospective studies.

## Conclusions

In conclusion, the AQP4-IgG immune response of our NMOSD cohort was directed against multiple epitopes of AQP4 in all analyzed serum samples, reflecting the heterogeneous properties of these autoantibodies. NMOSD sera showed two broad patterns of epitope recognition, a loop A-dependent pattern A and a loop A-independent pattern B. These patterns further revealed a change of AQP4-IgG epitope recognition during the course of disease in at least some of our NMOSD patients. Finally, different patterns in the serum versus CSF compartment suggest restricted intrathecal AQP4-IgG recognition in five out of six patients. Our results provide strong evidence that serum AQP4-IgGs are dependent on both defined extracellular epitopes and conformational arrangement.

## Abbreviations

AQP4, aquaporin-4; BBB, blood-brain barrier; CNS, central nervous system; CSF, cerebrospinal fluid; EmGFP, emerald green fluorescence protein; FACS; fluorescence activated cell sorting; HC, healthy control; HEK, human embryonic kidney; IPND, panel for NMOSD diagnosis; LETM, longitudinally extensive transverse myelitis; MFI, median fluorescence intensity; MS, multiple sclerosis; NMOSD, neuromyelitis optica spectrum disorders; OAPs, orthogonal arrays of particles; ON, optic neuritis; OND, other neurological diseases; rAbs, recombinant antibodies; WT, wildtype

## Additional files

Additional file 1: Primers for mutagenesis of human AQP4-M23 isoform. (PDF 46 kb) Additional file 2: Flow cytometry gating strategy. (PDF 287 kb) Additional file 3: (A) Binding of AQP4 specific monoclonal rAbs to AQP4-M1 versus AQP4-M23 isoforms, (B) Heatmap of rAbs against AQP4-M23, AQP4-M1 and AQP4-M23 mutants. (PDF 337 kb) Additional file 4: BN-page characterization of AQP4 wildtype and mutant proteins. (PDF 328 kb) Additional file 5: Heatmap of serum AQP4-antibody binding ratios against AQP4-M23, AQP4-M1 and AQP4-M23 mutants (columns) in baseline samples of 47 NMOSD patients. (PDF 88 kb) Additional file 6: Heatmap of serum AQP4-antibody binding (in percent of AQP4-M23) against AQP4-M23, AQP4-M1, and AQP4-M23 mutants (columns) in 49 follow-up samples of 20 NMOSD patients. (PDF 99 kb)
