# Supplementary material for: Characterization of the binding pattern of human aquaporin-4 autoantibodies in patients with neuromyelitis optica spectrum disorders
Source: J Neuroinflammation. 2016 Jul 1;13:176. doi: 10.1186/s12974-016-0642-3 (PMC4930584; doi:10.1186/s12974-016-0642-3)
Supplement: Additional file 1: — Primers for mutagenesis of human AQP4-M23 isoform. (PDF 46 kb) [file 12974_2016_642_MOESM1_ESM.pdf]

Additional File 1

Primer for mutagenesis of human AQP4-M23 isoform.

Myc-Tag insertion- hAQP4 Loop A (position G^{60})	Sense Primer: 5`-<br>TCAACTGGGGTGAGCAGAAGCTGATCTCTGAGGAAGA<br>CCTGGGAACAGAAAAGC-3’<br>Antisense Primer: 5`-<br>GCTTTTCTGTTCCCAGGTCTTCCTCAGAGATCAGCTTC<br>TGCTCACCCCAGTTGA-3’
Myc-Tag insertion- hAQP4 Loop C (position V^{141})	Sense Primer: 5`-<br>CCTCCCAGTGTGGAGCAGAAGCTGATCTCTGAGGAAG<br>ACCTGGTGGGAGGCCTG-3’<br>Antisense Primer: 5`-<br>CAGGCCTCCCACCAGGTCTTCCTCAGAGATCAGCTTC<br>TGCTCCACACTGGGAGG-3’
Myc-Tag insertion- hAQP4 Loop E (position G^{225})	Sense Primer: 5`-<br>GTTATCATGGGAGAGCAGAAGCTGATCTCTGAGGAAG<br>ACCTGAATTGGGAAAAC-3’<br>Antisense Primer: 5`-<br>GTTTTCCCAATTCAGGTCTTCCTCAGAGATCAGCTTCT<br>GCTCTCCCATGATAAC-3’
Delta A3 (LPVD → AAAA) loop A	Sense Primer: 5`-<br>GAACAGAAAAGCCTGCAGCGGCCGCCATGGTTCTCAT<br>CTC-3’<br>Antisense Primer: 5`-<br>GAGATGAGAACCATGGCGGCCGCTGCAGGCTTTTCTG<br>TTC-3’
N153Q loop C:	Sense Primer: 5`-

	ACCATGGTTCATGGACAACTTACCGCTGGT-3’<br><br>Antisense Primer: 5`-<br><br>ACCAGCGGTAAGTTGTCCATGAACCATGGT-3’
H230G loop E:	Sense Primer: 5`-<br><br>GGGAAATTGGGAAAACGGTTGGATATATTG-3’<br><br>Antisense Primer: 5`-<br><br>CAATATATCCAACCGTTTTCCCAATTTCCC-3’
