# Supplementary material for: Characterization of the binding pattern of human aquaporin-4 autoantibodies in patients with neuromyelitis optica spectrum disorders
Source: J Neuroinflammation. 2016 Jul 1;13:176. doi: 10.1186/s12974-016-0642-3 (PMC4930584; doi:10.1186/s12974-016-0642-3)
Supplement: Additional file 2: — Flow cytometry gating strategy. (PDF 287 kb) [file 12974_2016_642_MOESM2_ESM.pdf]

Additional File 2

Flow cytometry gating strategy.

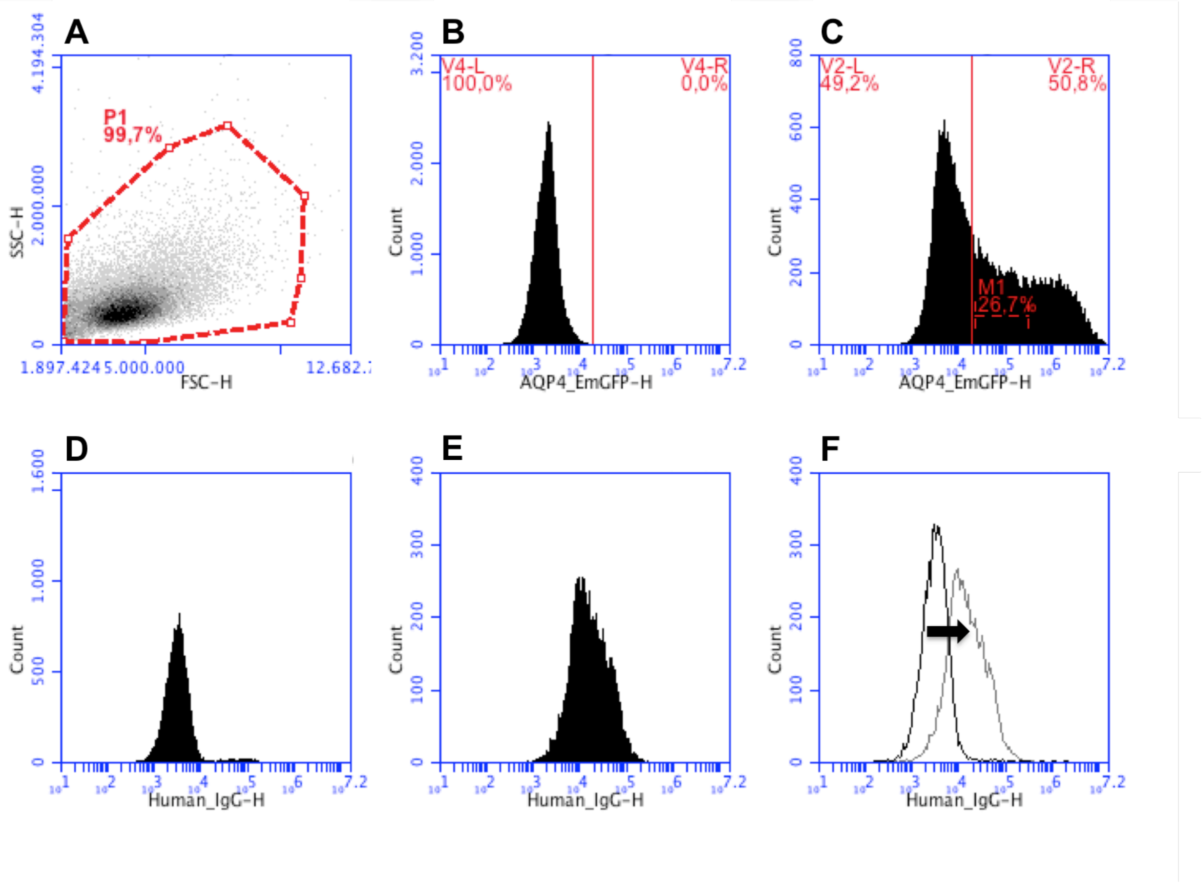

Gating and analysis strategy for AQP4 expressing HEK-293A cells. (A) Gating of the HEK-293A main cell population based on the FCS-H/SSC-H profile in the dot plot (P1). (B) Untransfected HEK-293A cells (< 10^4) were used as control for gating purpose (V4-L). (C) Gating of the AQP4-EmGFP negative (V2-L) and AQP4-EmGFP positive (V2-R) HEK-293A cell population, respectively. (D+E) Binding of serum human IgG antibodies to AQP4 results in a shift of the MFI when gated on AQP4-EmGFP positive cells (M1) compared to the MFI gated on AQP4-EmGFP negative cells (V2-L). (F) Overlay between an AQP4-IgG positive serum sample and HC shows a shift in the MFI (arrow) when gated on the AQP4-EmGFP positive cells (M1). FSC-H=forward scatter (height). SSC-H=side scatter (height). (Em)GFP-A=(emerald) green fluorescent protein (area). MFI=median fluorescence intensity. HC=healthy control.
