# Supplementary material for: Characterization of the binding pattern of human aquaporin-4 autoantibodies in patients with neuromyelitis optica spectrum disorders
Source: J Neuroinflammation. 2016 Jul 1;13:176. doi: 10.1186/s12974-016-0642-3 (PMC4930584; doi:10.1186/s12974-016-0642-3)
Supplement: Additional file 3: — (A) Binding of AQP4 specific monoclonal rAbs to AQP4-M1 versus AQP4-M23 isoforms, (B) Heatmap of rAbs against AQP4-M23, AQP4-M1 and AQP4-M23 mutants. (PDF 337 kb) [file 12974_2016_642_MOESM3_ESM.pdf]

Additional File 3

(A) Binding of AQP4 specific monoclonal rAbs to AQP4-M1 versus AQM4-M23 isoforms.

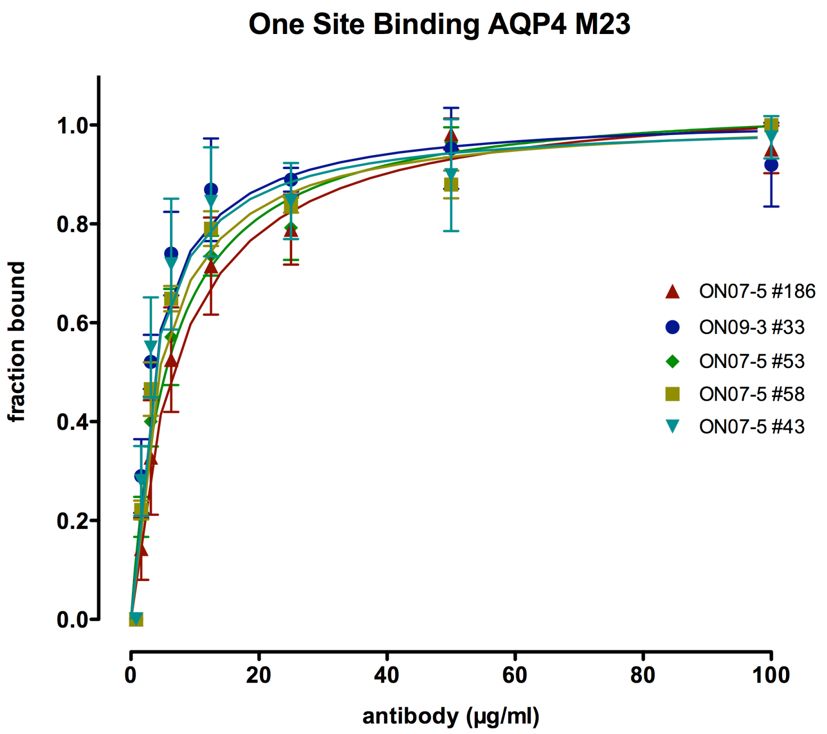

Binding curves for the 5 human recombinant AQP4-IgG antibodies provided by Dr. Jeffrey Bennerr (mean with standard deviation). Curves represent fits to single-site binding model.

(B) Heatmap of rAbs against AQP4-M23, AQP4-M1 and AQP4-M23 mutants.

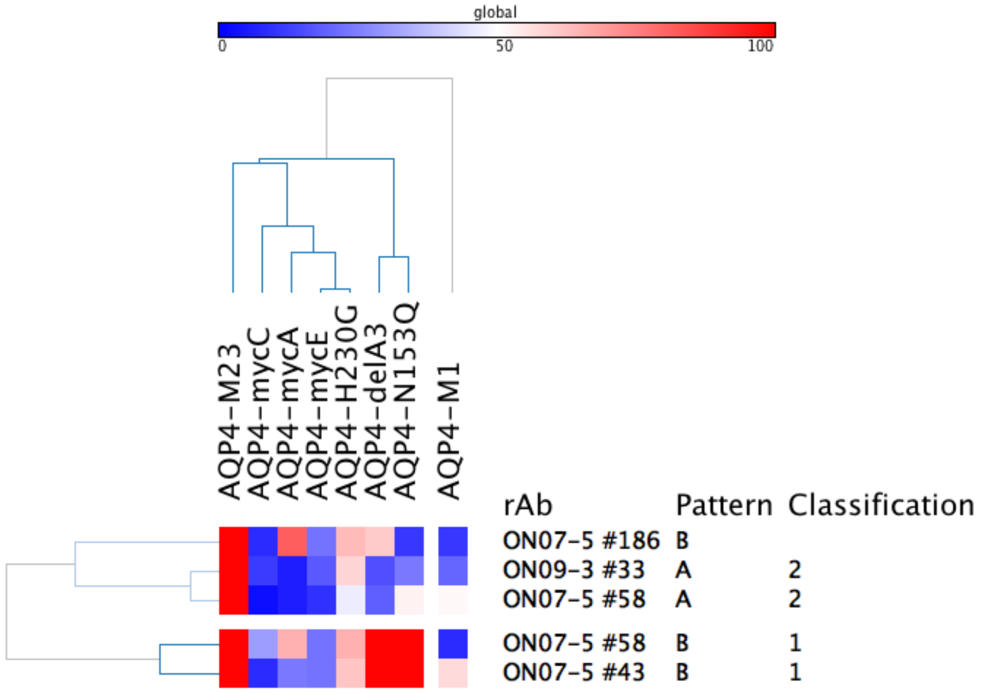

Heatmap of rAbs against AQP4-M23, AQP4-M1 and AQP4-M23 mutants (columns). Data are shown as percent binding of AQP4-M23. Values range from blue (0%) to white (50%) to red (100%). Columns were clustered according to their Pearson's correlation coefficients and rows were cluster according to their Eucledian distance (both complete linkage). Two major antibody binding patterns were identified, a loop-A dependent pattern A and an independent pattern B. These patterns are according to the classification described by Owens et al in 2015. The heatmap was generated using GENE-E matrix visualization and analysis software (http://www.broadinstitute.org/cancer/software/GENE-E/index.html).
