# Supplementary material for: Characterization of the binding pattern of human aquaporin-4 autoantibodies in patients with neuromyelitis optica spectrum disorders
Source: J Neuroinflammation. 2016 Jul 1;13:176. doi: 10.1186/s12974-016-0642-3 (PMC4930584; doi:10.1186/s12974-016-0642-3)
Supplement: Additional file 4: — BN-page characterization of AQP4 wildtype and mutant proteins. (PDF 328 kb) [file 12974_2016_642_MOESM4_ESM.pdf]

Additional File 4

BN page characterization of AQP wildtype and mutant proteins.

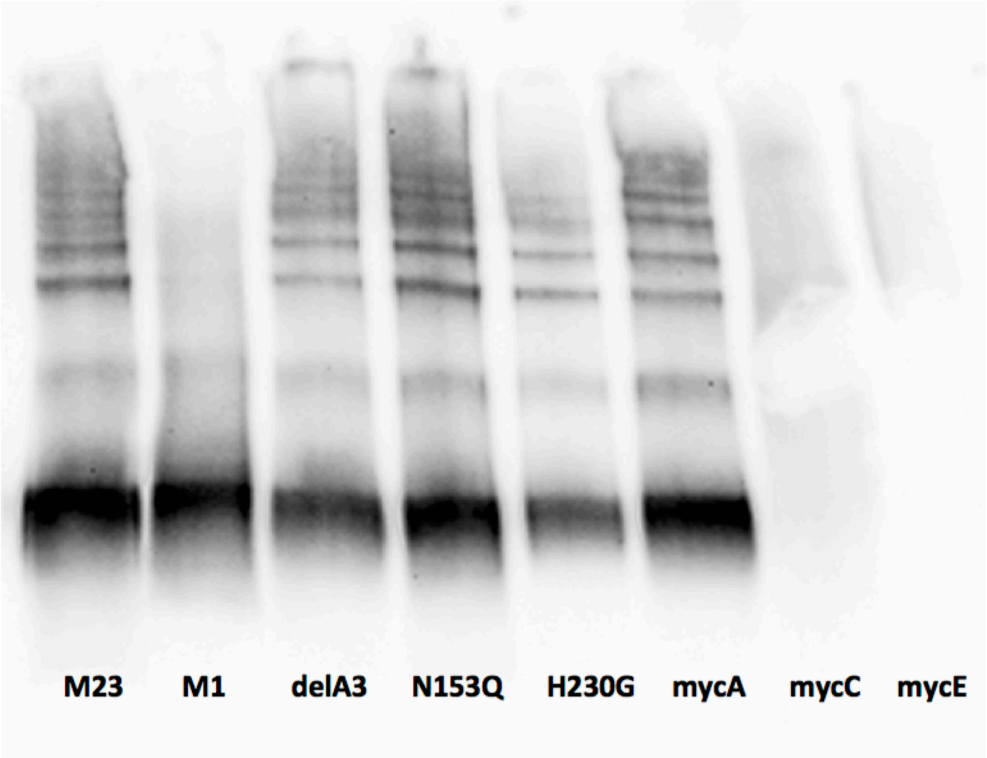

The formation of supramolecular OAPs was visualized by BN-PAGE showing the effects of extracellular loop mutations. AQP4-M23 as well as mutant constructs delA3, N153Q, H230G and mycA show the presence of large aggregates, whereas higher order arrays are absent in AQP4-M1 mycC and mycE mutant constructs.
