# Supplementary material for: Characterization of the binding pattern of human aquaporin-4 autoantibodies in patients with neuromyelitis optica spectrum disorders
Source: J Neuroinflammation. 2016 Jul 1;13:176. doi: 10.1186/s12974-016-0642-3 (PMC4930584; doi:10.1186/s12974-016-0642-3)
Supplement: Additional file 5: — Heatmap of serum AQP4-antibody binding ratios against AQP4-M23, AQP4-M1 and AQP4-M23 mutants (columns) in baseline samples of 47 NMOSD patients. (PDF 88 kb) [file 12974_2016_642_MOESM5_ESM.pdf]

Additional File 5

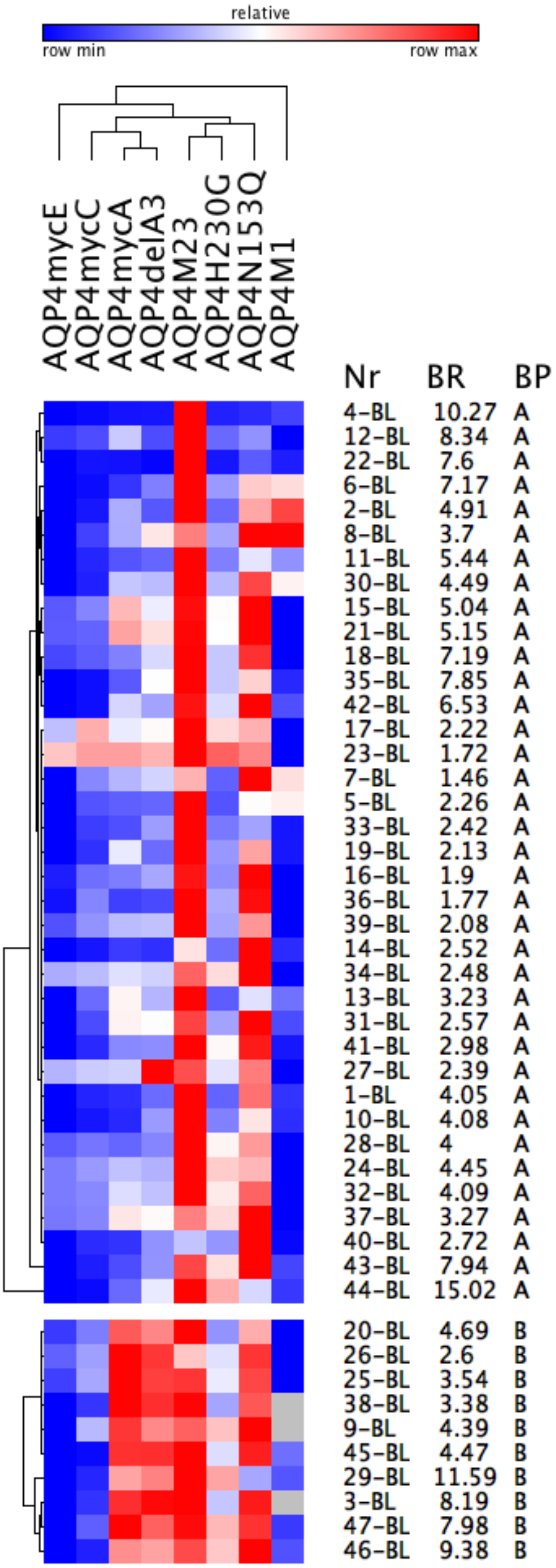

Heatmap of serum AQP4-antibody binding ratios against AQP4-M23, AQP4-M1 and AQP4-M23 mutants (columns) in baseline samples of 47 NMOSD patients. Rows are individual samples with patient IDs (Nr), FACS AQP4-M23 binding ratios (BR) and AQP4-IgG binding patterns (BP) shown at the right side. Data are shown as absolute FACS binding ratios. Values range from blue (row minimum) to red (row maximum) of each row. Columns were clustered according to their Pearson's correlation coefficients and rows were cluster according to their Eucledian distance (both average linkage). Also with this method two major antibody binding patterns were identified, a loop-A dependent pattern A and an independent pattern B. The heatmap was generated using GENE-E matrix visualization and analysis software (http://www.broadinstitute.org/cancer/software/GENE-E/index.html).
