# Supplementary material for: Characterization of the binding pattern of human aquaporin-4 autoantibodies in patients with neuromyelitis optica spectrum disorders
Source: J Neuroinflammation. 2016 Jul 1;13:176. doi: 10.1186/s12974-016-0642-3 (PMC4930584; doi:10.1186/s12974-016-0642-3)
Supplement: Additional file 6: — Heatmap of serum AQP4-antibody binding (in percent of AQP4-M23) against AQP4-M23, AQP4-M1, and AQP4-M23 mutants (columns) in 49 follow-up samples of 20 NMOSD patients. (PDF 99 kb) [file 12974_2016_642_MOESM6_ESM.pdf]

**Additional File 6**

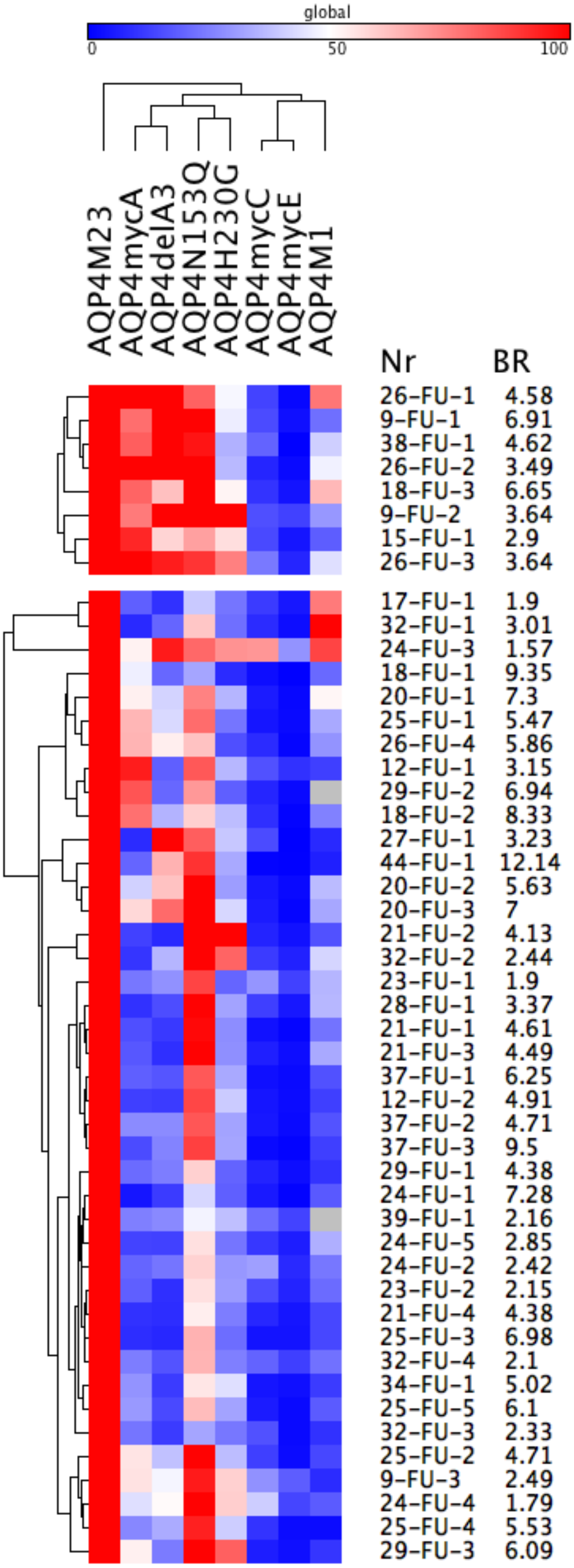


| Nr | BR | BP |
|---|---|---|
| 26-FU-1 | 4.58 | B |
| 9-FU-1 | 6.91 | B |
| 38-FU-1 | 4.62 | B |
| 26-FU-2 | 3.49 | B |
| 18-FU-3 | 6.65 | B |
| 9-FU-2 | 3.64 | B |
| 15-FU-1 | 2.9 | B |
| 26-FU-3 | 3.64 | B |
| 17-FU-1 | 1.9 | A |
| 32-FU-1 | 3.01 | A |
| 24-FU-3 | 1.57 | A |
| 18-FU-1 | 9.35 | A |
| 20-FU-1 | 7.3 | A |
| 25-FU-1 | 5.47 | A |
| 26-FU-4 | 5.86 | A |
| 12-FU-1 | 3.15 | A |
| 29-FU-2 | 6.94 | A |
| 18-FU-2 | 8.33 | A |
| 27-FU-1 | 3.23 | A |
| 44-FU-1 | 12.14 | A |
| 20-FU-2 | 5.63 | A |
| 20-FU-3 | 7 | A |
| 21-FU-2 | 4.13 | A |
| 32-FU-2 | 2.44 | A |
| 23-FU-1 | 1.9 | A |
| 28-FU-1 | 3.37 | A |
| 21-FU-1 | 4.61 | A |
| 21-FU-3 | 4.49 | A |
| 37-FU-1 | 6.25 | A |
| 12-FU-2 | 4.91 | A |
| 37-FU-2 | 4.71 | A |
| 37-FU-3 | 9.5 | A |
| 29-FU-1 | 4.38 | A |
| 24-FU-1 | 7.28 | A |
| 39-FU-1 | 2.16 | A |
| 24-FU-5 | 2.85 | A |
| 24-FU-2 | 2.42 | A |
| 23-FU-2 | 2.15 | A |
| 21-FU-4 | 4.38 | A |
| 25-FU-3 | 6.98 | A |
| 32-FU-4 | 2.1 | A |
| 34-FU-1 | 5.02 | A |
| 25-FU-5 | 6.1 | A |
| 32-FU-3 | 2.33 | A |
| 25-FU-2 | 4.71 | A |
| 9-FU-3 | 2.49 | A |
| 24-FU-4 | 1.79 | A |
| 25-FU-4 | 5.53 | A |
| 29-FU-3 | 6.09 | A |

Heatmap of serum AQP4-antibody binding (in percent of AQP4-M23) against AQP4-M23, AQP4-M1 and AQP4-M23 mutants (columns) in 49 follow-up samples of 20 NMOSD patients. Rows are individual samples with patient IDs (Nr), FACS AQP4-M23 binding ratios (BR) and AQP4-IgG binding patterns (BP) shown at the right side. Data are shown as percent binding of AQP4-M23. Values range from blue (0%) to white (50%) to red (100%). Columns were clustered according to their Pearson's correlation coefficients and rows were cluster according to their Eucledian distance (both average linkage). Also with this validation cohort two major antibody binding patterns were confirmed, a loop-A dependent pattern A and an independent pattern B. The heatmap was generated using GENE-E matrix visualization and analysis software (http://www.broadinstitute.org/cancer/software/GENE-E/index.html).
